# Supplementary material for: Developmental Neurotoxicity of Trichlorfon in Zebrafish Larvae
Source: Int J Mol Sci. 2023 Jul 4;24(13):11099. doi: 10.3390/ijms241311099 (PMC10342510; doi:10.3390/ijms241311099)
Supplement: Supplementary file 1 [file ijms-24-11099-s001.zip › ijms-2481919-supplementary.pdf]

Figure legends

Figure S1. (A–C) Typical malformations images of 144 hpf zebrafish larvae after exposure to different concentrations of trichlorfon (0, 0.1, 2 amd 5 mg/L). SC: spinal curvature; YSE: yolk sac edema; TC: tail curvature.

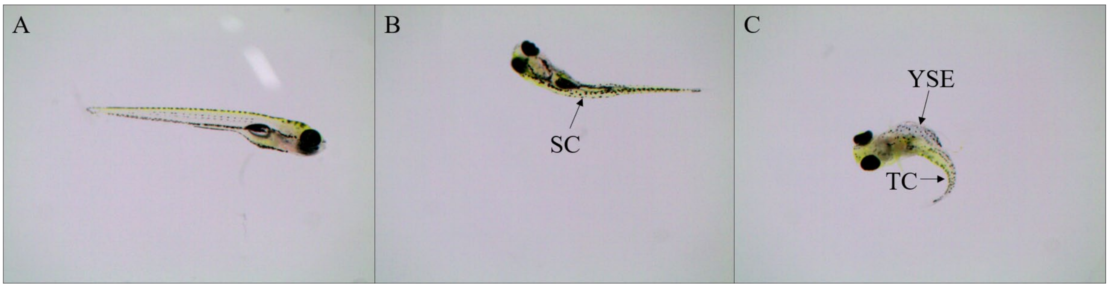

Table S1 Sequences of primers of genes used in the present study

Gene	Forward primer 5'-3'	Reverse primer 5'-3'	Accession No.
β-actin	ACAGGGAAAAGATGACACAGATCA	CAGCCTGGATGGCAACGTA	AF025305
α1-tubulin	AATCACCAATGCTTGCTTCGAGCC	TTCACGTCTTTGGGTACCACGTCA	NM_194388
mbp	AATCAGCAGGTTCTTCGGAGGAGA	AAGAAATGCACGACAGGGTTGACG	AY860977
syn2a	GTGACCATGCCAGCATTTC	TGGTTCTCCACTTTCACCTT	NM_001002597
shha	GCAAGATAACGCGCAATTCGGAGA	TGCATCTCTGTGTCATGAGCCTGT	DRU30711
gap-43	TGCTGCATCAGAAGAACTAA	CCTCCGGTTTGATTCCATC	NM_131341
ache	CATACGCACAATACGCTGCC	TACACAGCACCATGCGAGTT	NM_131846
chrna7	CCGGCAACATCTGACTCTGT	CAGTTCAACAGCACCACACG	NM_201219
chata	ACCGATGGTACGACAAACCC	AGAGTGTTCACAGACGACGC	NM_001130719
hact	CTCTCGAACCCGGCTGTATC	TATCTTCCCAAGCCATGCGG	XM_021473914
vacht	TACTGTATGAGTTCGCGGGC	AAGGGCTTGAGCACAGTCAG	NM_001077550
manf	AGATGGAGAGTGTGAAGTCTGTGTG	CAATTGAGTCGCTGTCAAACTTG	NM_001076629
bdnf	ATAGTAACGAACAGGATGG	GCTCAGTCATGGGAGTCC	NM_131595
nr4a2b	AGGCTAGAGGATCTCCGTCC	GCACCGTGCGCTTAAAGAAT	NM_001002406
drd2b	GATCTCCGTTGTTTGGGTGC	GCGGGGTTGGCAATTTCAC	NM_197936
drd4a	TGTTCGGCATCAACAACGTC	ACATTCCGCAGTACAGGAGC	NM_001012616
drd4b	AGCATCTCCTGTCATCTTCGG	CAGCATGAGCATAATGGGGC	NM_001012618
drd7	GATCTCCGTTGTTTGGGTGC	GCGGGGTTGGCAATTTCAC	NM_001113643

tph1	TCTGTGAACTCTACGTGTGG	CACTGGGAGCATCAGACG	AF548566
tph2	ATCCATCCTTGCTCTCCAAC	TCTGTGAACTCTACGTGTGG	NM_214795
tphr	AGATCCCATACCACACGTAGAG	CGGTTCAGGAGTGTAAAGAGG	AB125219
serta	ACCACCAGAGTCCTAAATGTTCCAG	CTCTTCCTTCATCTGTGTGCCTTCC	NM_001039972
sertb	AACCCTAACAGCAGTCCTCA	GGCCTCACCGTCACACAATA	NM_001177459
htrlaa	ATGAGGATGAGCGGGATGTAG	CAATCAGCCAGGACCACG	NM_001123321
htrlab	CTGTGTCGCCTGCACTTTTC	TGATCTCCAAAGACTCGCCG	NM_001145766
